# Unsolved mysteries: How does lipid peroxidation cause ferroptosis?

**DOI:** 10.1371/journal.pbio.2006203

**Published:** 2018-05-24

**Authors:** Huizhong Feng, Brent R. Stockwell

**Affiliations:** 1 Department of Biological Sciences, Columbia University, New York, New York, United States of America; 2 Department of Chemistry, Columbia University, New York, New York, United States of America

## Abstract

Ferroptosis is a cell death process driven by damage to cell membranes and linked to numerous human diseases. Ferroptosis is caused by loss of activity of the key enzyme that is tasked with repairing oxidative damage to cell membranes—glutathione peroxidase 4 (GPX4). GPX4 normally removes the dangerous products of iron-dependent lipid peroxidation, protecting cell membranes from this type of damage; when GPX4 fails, ferroptosis ensues. Ferroptosis is distinct from apoptosis, necroptosis, necrosis, and other modes of cell death. Several key mysteries regarding how cells die during ferroptosis remain unsolved. First, the drivers of lipid peroxidation are not yet clear. Second, the subcellular location of lethal lipid peroxides remains an outstanding question. Finally, how exactly lipid peroxidation leads to cell death is an unsolved mystery. Answers to these questions will provide insights into the mechanisms of ferroptotic cell death and associated human diseases, as well as new therapeutic strategies for such diseases.

## Introduction

**Fig 1:**
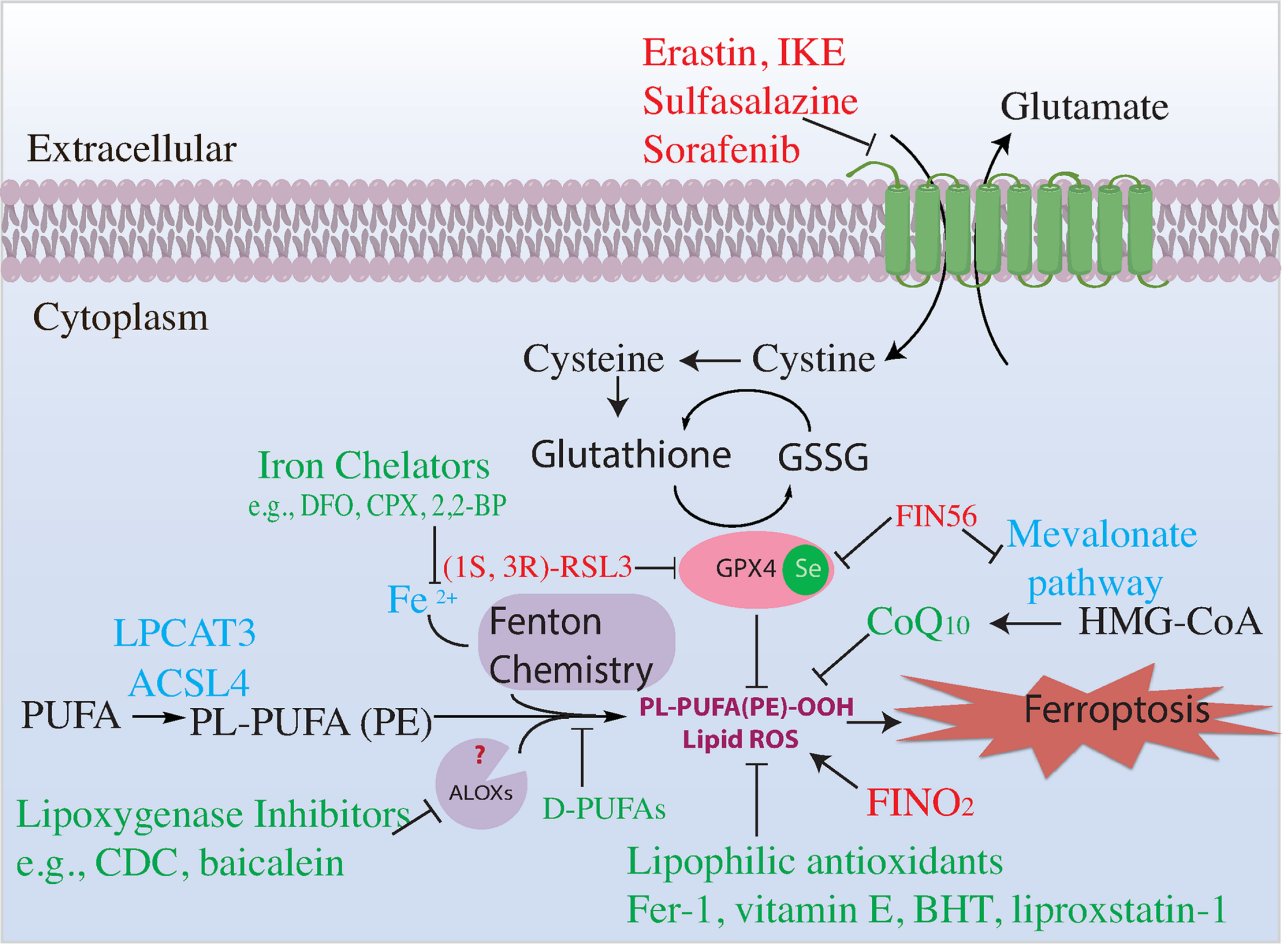
Pathways regulating ferroptosis. Summary of ferroptosis mechanisms and signaling pathway. Ferroptosis inducers/sensitizers are colored red. Ferroptosis inhibitors are colored green. 2,2-BP, 2,2-bipyridyl; ACSL4, acyl-CoA synthetase long chain family member 4; ALOX, arachidonate lipoxygenase; BHT, butylated hydroxytoluene; CoQ_10_, coenzyme Q_10_; CPX, ciclopirox olamine; DFO, deferoxamine; D-PUFA, deuterated polyunsaturated fatty acids; Fer-1, ferrostatin-1; FIN56, ferroptosis inducer 56; FINO_2_, ferroptosis inducer endoperoxide; GPX4, glutathione peroxidase 4; GSSG, glutathione disulfide; HMG-CoA, β-hydroxy β-methylglutaryl-CoA; IKE, imidazole ketone erastin; LPCAT3, lysophosphatidylcholine acyltransferase 3; PL-PUFA (PE), polyunsaturated-fatty-acid-containing phospholipids; PL-PUFA(PE)-OOH, polyunsaturated-fatty-acid-containing-phospholipid hydroperoxides; PUFA, polyunsaturated fatty acid; ROS, reactive oxygen species; RSL3, RAS-selective lethal 3

The fundamental building block of life is the cell, the smallest living unit within multicellular organisms. Cells, like the organisms they constitute, live and die. According to the recommendations of the Nomenclature Committee on Cell Death (NCCD), cell death can be accidental or regulated [1]. Accidental cell death occurs when cells experience overwhelming physical, chemical, or mechanical insults; such accidental cell death cannot be modulated by molecularly targeted interventions. In contrast, regulated cell death can be modulated pharmacologically and genetically, as it is controlled by molecular mechanisms. The NCCD defines programmed cell death to be a subset of regulated cell death that occurs in normal physiological contexts [1]. Caspase-dependent apoptosis is a well-known form of regulated, programmed cell death. Ferroptosis is a recently described form of cell death that is regulated [2], in the sense of the NCCD definition, as it can be enhanced and suppressed by specific genetic and pharmacological interventions. Ferroptosis is characterized by loss of activity of glutathione peroxidase 4 (GPX4), resulting in overwhelming accumulation of lethal lipid peroxides [3] (Fig 1).

Ferroptosis was originally coined as a term for the unique form of cell death initated by the small molecules erastin and RAS-selective lethal 3 (RSL3) [3] and is now defined as a form of cell death that involves accumulation of lipid peroxides and that is suppressed by iron chelators and lipophilic antioxidants [2]. Other compounds that induce cytosolic or mitochondrial reactive oxygen species (ROS) do not induce ferroptosis [3,4]; thus, general ROS production is not related to ferroptosis. In contrast, ferroptosis is tightly linked to lipid peroxidation and can be thought of as death by lipid peroxidation. An open question is whether any type of lethal lipid peroxidation is classified as ferroptosis or whether only certain types of lethal lipid peroxidation should be termed ferroptosis. Since the current definition of ferroptosis is a cell death process involving lipid peroxidation that is suppressed by both iron chelators and lipophilic antioxidants, any lethal iron-dependent lipid peroxidation would be classified as ferroptosis. However, just as elucidation of the mechanisms driving apoptosis has revealed different pathways leading to a similar endpoint, such as the instrinsic and extrinsic apoptotic pathways, it may be that ferroptosis can be initiated and executed by distinct pathways involving different types of lethal iron-dependent lipid peroxidation. More details on the specific mechanisms involved in lethal lipid peroxidation will be needed to resolve this question.

Ferroptosis has been implicated in numerous human pathologies and therapeutic strategies, but a normal physiological function for ferroptosis has not been identified, except perhaps as a tumor suppression mechanism. A recent review summarized the evidence for ferroptosis in models of a variety of degenerative diseases of the kidney, heart, liver, and brain, including models of Parkinson, Huntington, and Alzheimer diseases, dementia, and traumatic and hemorrhagic injuries [2].

Pathologies involving ferroptosis have also been found in contexts in which iron is abundant, such as in red blood cells. Indeed, ferroptosis has been implicated in the complications of blood transfusions. A recent study found that transfusion of storage-damaged red blood cells induces a macrophage-dependent inflammatory response by Ly6C^hi^ monocytes [5]. Moreover, macrophages underwent ferroptosis following phagocytosis of storage-damaged red blood cells. This suggests that inhibitors of ferroptosis might improve outcomes of blood transfusion. Ferroptosis has also been linked to the iron-overload disease liver hemochromatosis [2].

As noted above, there are few reports of natural functions of ferroptosis. However, the p53 tumor suppressor has been suggested to use ferroptosis as a tumor suppression mechanism [6,7], suggesting that ferroptosis may have a natural function in suppressing the developing of some tumors in mammals.

Ferroptosis also may be useful for eliminating cancer cells that have become dependent on suppression of ferroptosis for their surivival. Two recent studies found that some of the most drug-resistant and hard-to-kill cancer cells—namely, those that have undergone epithelial-to-mesenchymal transition [8] and those that persist after conventional chemotherapy or targeted therapy [9]—are highly sensitive to GPX4 inhibitors and ferroptosis in general. This suggests that ferroptosis-inducing agents might be developed as a new class of cancer therapies.

Finally, ferroptosis may be involved in the toxic effects of environmental contaminants. A recent report found that mice fed arsenite in their drinking water exhibited increased neuronal ferroptosis [10]. Thus, ferroptosis markers may aid in detecting individuals exposed to arsenite in their drinking water, and ferroptosis inhibitors might be useful for such communities.

Four ways of initiating ferroptosis have been discovered (Table 1) [3]. Class 1 ferroptosis inducers act by starving cells of the amino acid cysteine. Such compounds act by inhibiting system x_c_^−^, a transmembrane cystine-glutamate antiporter, which imports cystine (the oxidized, disulfide form of cysteine) into cells. When this process is blocked, cysteine is depleted from these cells.

**Table 1:** Ferroptosis inducers.

Class	Class Characteristics	Impact on Ferroptosis	Compound Examples	Suitable for in vivo use
Class 1	Inhibition of system x_c_^−^	Prevents cystine import, causes GSH depletion and loss of GPX4 activity	Erastin, PE, IKE, other erastin analogs, sulfasalazine, sorafenib, glutamate	PE, IKE, sorafenib
Class 2	Direct inhibition of GPX4	Covalently interacts with GPX4 and inhibits its enzymatic activity	RSL3, ML162, DPI compounds 7,10,12,13,17,18,19	No
Class 3	Depletion of GPX4 protein and CoQ_10_	Depletes GPX4 protein and simultaneously causes depletion of CoQ_10_ via SQS-mevalonate pathway	FIN56 and CIL56	Unknown
Class 4	Induction of lipid peroxidation	Oxidizes iron, drives lipid peroxidation and indirect inactivation of GPX4	FINO_2_	Unknown
Others: BSO, DPI2, cisplatin, cysteinase, statins, ferric ammonium citrate, trigonelline, CCI_4_, silica-based nanoparticles, nonthermal plasma				

Abbreviations: BSO, buthionine sulfoximine; CCI_4_, carbon tetrachloride; CIL56, caspase-independent lethal 56; CoQ_10_, coenzyme Q_10_; DPI, diverse pharmacological inhibitor; FIN56, ferroptosis inducer 56; FINO_2_, ferroptosis inducer endoperoxide; GPX4, glutathione peroxidase 4; GSH, glutathione; IKE, imidazole ketone erastin; ML162, Molecular Libraries 162; PE, piperazine erastin; RSL3, RAS-selective lethal 3; SQS, squalene synthase

Cysteine has a number of functions in cells—most importantly, in the context of ferroptosis, as a building block for the biosynthesis of glutathione (GSH). GSH is a cofactor and substrate for GPX4 and is required for the lipid repair function of this enzyme. Depletion of GSH through cysteine starvation leads to loss of GPX4 activity, resulting in accumulation of unrepaired lipid peroxides and death by ferroptosis.

Erastin and its more potent analogs imidazole ketone erastin (IKE) and piperazine erastin (PE), as well as the FDA-approved drugs sulfasalazine and sorafenib, are small molecule class 1 ferroptosis inducers. In addition, high extracellular concentrations of the amino acid neurotransmitter glutamate can act as a class 1 ferroptosis inducer. All of these are suitable for use in vitro, although, as sorafenib and glutamate also activate other nonferroptotic mechanisms, they must be used with care as chemical probes of ferroptosis. Erastin has low solubiity and poor metabolic stability and pharmacokinetics, preventing its use in vivo. Likewise, sulfasalazine has low metabolic stability and low potency, preventing its reliable use in vivo. However, sorafenib, IKE, and PE are suitable for in vivo use, as they have high potency, sufficient metabolic stability, and acceptable pharmacokinetic profiles.

Class 2 ferroptosis inducers act through direct inhibition of GPX4. RSL3 (also known as [1S,3R]-RSL3 to indicate the stereochemical configuration) and a number of other reported compounds [11] covalently interact with the nucleophilic active-site selenocyteine of GPX4 and inhibit its enzymatic activity, resulting in loss of its lipid repair function, accumulation of lethal lipid peroxides, and consequent cell death [12]. RSL3 is primarily suitable for in vitro use as a potent and selective inhibitor of GPX4; its inactive stereoisomer (1R, 3R)-RSL3 is a useful inactive negative control compound. Unfortunately, none of the class 2 ferroptosis inducers are suitable for in vivo use, due to low solubility and difficulty characterizing their pharmacokinetics. However, numerous genetic studies in vivo have examined the effects of loss of the *gpx4* gene.

Class 3 ferroptosis inducers, which include the compounds ferroptosis inducer 56 (FIN56) and caspase-independent lethal 56 (CIL56), act by depleting GPX4 protein from cells and simultaneously causing depletion of mevalonate-derived coenzyme Q_10_ (CoQ_10_), which functions as an endogenous lipophilic antioxidant in addition to its well-known function in the mitochondrial electron transport chain [13]. CIL56 also activates a distinct necrotic cell death mechanism, whereas FIN56 is a specific inducer of ferroptosis; thus, FIN56 is the more specific chemical probe for in vitro use. Neither compound has been used in vivo.

The only class 4 ferroptosis inducer currently known is ferroptosis inducer endoperoxide (FINO_2_), which acts by oxidizing iron, indirectly inactivating GPX4, and further driving lipid peroxidation [14,15]. This compound is suitable for use in vitro and has not been evaluated in vivo, although its moderate potency suggests it might not be effective in vivo. A recent report suggested that nonthermal plasma may be able to similarly redox cycle iron and perhaps induce ferroptosis through this class 4 mechanism [16].

Blocking ferroptosis may be useful in treating some degenerative diseases. A number of ferroptosis inhibitors that could be used for such purposes have been reported (Table 2). The first two types of ferroptosis inhibitors identified, as noted above, were iron chelators—such as deferoxamine and ciclopirox—and lipophilic antioxidants—such as α-tocopherol (a component of vitamin E), butylated hydroxytoluene (BHT), ferrostatin-1 (Fer-1), and liproxstatin-1. Iron chelators prevent the initiation of lipid peroxidation by inhibiting lipoxygenases (LOXs) and the propagation of lipid peroxidation by suppressing Fenton chemistry. Deferoxamine and ciclopirox are suitable for in vivo use; both are drugs approved for human use. Lipophilic antioxidants likely exert their effects through a radical trapping mechanism to suppress lipid peroxidation [17] [18]. In addition, other ferroptosis inhibitors have been identified, including deuterated polyunsaturated fatty acids (D-PUFAs), inhibitors of acyl-CoA synthetase long chain family member 4 (ACSL4), glutaminolysis inhibitors, LOX inhibitors, cycloheximide, beta-mercaptoethanol, dopamine, selenium, and vildagliptin [2]. Table 2 summarizes the utility of these compounds in vitro and in vivo.

**Table 2:** Ferroptosis inhibitors.

Class	Class Characteristics	Impact on Ferroptosis	Compound Examples	Suitable for In Vivo Use
Class 1	Iron chelators	Deplete iron and prevent iron-dependent lipid peroxidation	Deferoxamine, cyclipirox, deferiprone	Yes
Class 2	Lipophilic antioxidants	Prevent lipid peroxidation	Vitamin E, BHT, Fer-1, liproxstatin-1, XJB-5-131, CoQ_10_	XJB-5-131
Class 3	D-PUFAs	Prevents initiation and propagation of lipid peroxidation	D_4_-arachidonic acid, D_10_-docosahexaenoic acid	Yes
Class 4	LOX inhibitors	Inactivate LOX and block LOX-induced lipid peroxidation	CDC, baicalein, PD-146176, AA-861, zileuton	Not sufficiently selective in most cases
Others: glutaminolysis inhibitors, cycloheximide, beta-mercaptoethanol, dopamine, selenium, vildagliptin.				

Abbreviations: AA, arachidonic acid; BHT, butylated hydroxytoluene; CoQ_10_, coenzyme Q_10_; D-PUFA, deuterated polyunsaturated fatty acid; Fer-1, ferrostatin-1; LOX, lipoxygenase

Several studies have examined which lipids undergo peroxidation during ferroptosis. Lipidomic studies revealed that polyunsaturated-fatty-acid-containing phospholipids (PUFA-PLs) are the lipids most susceptible to peroxidation and drive the subsequent cell death (Fig 1). Bis-allylic carbons (carbon atoms that are adjacent to two neighboring carbon–carbon double bonds) in such PUFA-PLs are chemically susceptible to attack by radicals, LOXs, and oxygen, making these the key positions within lipids that drive ferroptosis [19]. PUFA-PLs are the most vulnerable lipid species, due to the presence of these bis-allylic sites in the membrane environment. It is worth noting that for death to proceed, free PUFAs must be incorporated into phospholipids by ACSL4 and lysophosphatidylcholine acyltransferase 3 (LPCAT3) [20,21] (Table 3).

**Table 3:** Key ferroptosis-related genes.

Gene	Name	Function
ACSL4	Acyl-CoA synthetase long chain family member 4	Converts free fatty acids into fatty CoA ester, required for ferroptosis
ALOXs	Arachidonate lipoxygenases	Catalyze peroxidation of AAs (PUFAs)
GPX4	Glutathione peroxidase 4	Reduces LOOHs in membrane phospholipids to suppress ferroptosis
LPCAT3	Lysophosphatidylcholine acyltransferase 3	Involved in biosynthesis of phospholipids, required for ferroptosis

Abbreviations: AA, arachidonic acid; LOOH, lipid hydroperoxide; PUFA, polyunsaturated fatty acid

There are several key outstanding questions in the lipid peroxidation process that drives ferroptosis. First, what causes lipid peroxidation—nonenzymatic pathways, i.e., Fenton chemistry, or enzymatic processes, such as LOXs? Second, what is the location in which lipid peroxidation takes place during ferroptosis? Does this event occur in the plasma membrane, mitochondria, endoplasmic reticulum (ER), lysosomes, and/or other subcellular locations? Third, how does lipid peroxidation lead to ferroptotic cell death? Does this stem from damage to specific membranes or through downstream generation of reactive products of lipid peroxidation? Here, we discuss each of these three unsolved mysteries and provide insight on how these mysteries might be solved.

## What causes lipid peroxidation: Fenton chemistry or LOXs?

**Fig 2:**
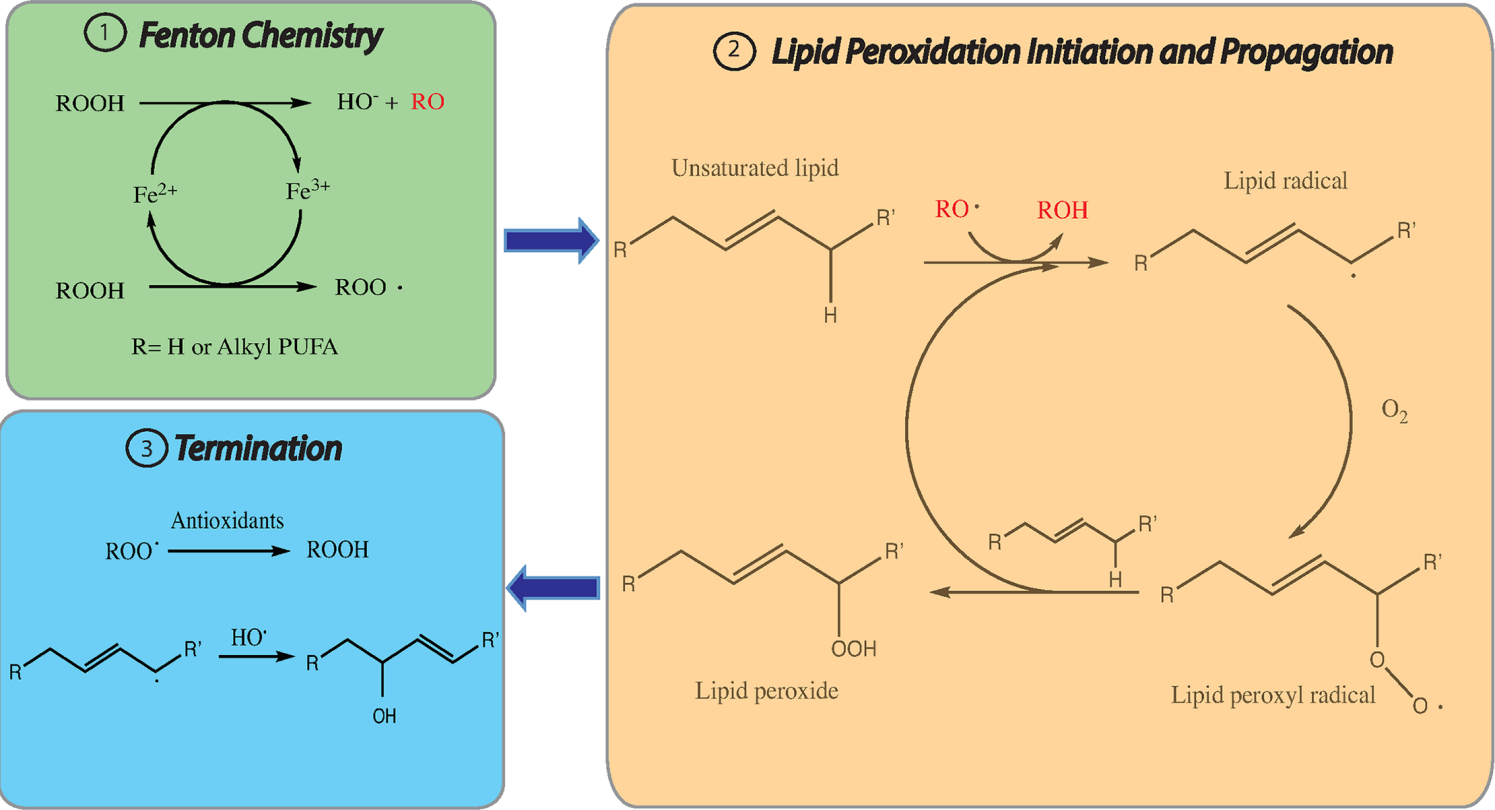
Fenton chemistry and lipid peroxidation in ferroptosis. There are three steps involved in nonenzymatic lipid peroxidation. The first step is the generation of lipid radicals (initiation). The second step is the creation of new lipid radicals (propagation). The final step is termination, either by antioxidants or another radical. PUFA, polyunsaturated fatty acid.

Oxidative damage to PUFA-PLs can take place via two mechanisms: the nonenzymatic free-radical chain reaction involving Fenton chemistry and enzymatic processes, most notably LOXs. Fenton chemistry refers to a series of reactions between peroxides and divalent ferrous salts to produce oxygen-centered radicals [22]. Under normal conditions, cellular iron is under precise regulation. However, there is a pool of loosely chelated iron in cells: while most cellular iron is bound to heme, there is soluble and chelatable ferrous iron in the cytoplasm which forms the labile iron pool [23]. This labile iron pool is the likely source of Fenton chemistry that generates hydroxyl and peroxyl radicals that are able to abstract hydrogen atoms from bis-allylic carbons of PUFAs and then cause peroxidation of PUFA-PLs (Fig 2). These reactions can be terminated by antioxidants and radicals [24]. Recent studies found that numerous inhibitors of ferroptosis—including Fer-1, liproxstatin-1, and numerous LOX inhibitors—act as radical trapping antioxidants to prevent the autooxidation and nonenzymatic destruction of membrane PUFA-PLs likely driven by Fenton chemistry during ferroptosis [25,26]. Moreover, addition of excess iron to cells sensitizes them to ferroptosis [3].

There are three well-defined classes of lipid oxidation enzymes: cyclooxygenases (COXs), cytochrome p450 (CYPs), and LOXs, among which LOX enzymes have been found to be the most important for ferroptosis. LOXs are a family of nonheme, iron-containing enzymes that catalyze dioxygenation of PUFAs [27]. In the case of arachidonic acid (AA), there are 6 arachidonate lipoxygenase (ALOX) genes in humans: *ALOX5*, *ALOX12*, *ALOX12B*, *ALOX15*, *ALOX15B*, *and ALOXE3* [12]. The 5-lipoxygenase enzyme, encoded by the *ALOX5* gene, oxidizes AA at carbon 5 and then forms 5-hydroperoxyeicosatetraenoic acid (5-HPETE). The 12- and 15- lipoxygenases are encoded by *ALOX12/ALOX12B and ALOX15/ALOX15B*, respectively, and are able to convert AA to 12-HPETE and 15-HPETE [28].

The mechanisms by which LOXs drive ferroptotic cell death, and the isoforms that drive this process, remain elusive. Several hypotheses have been proposed. One research group found that in mouse embryonic fibroblasts (MEF), 12/15-lipoxygenase-deficient cells were resistant to the lethality normally caused by GSH depletion using the GSH biosynthesis inhibitor buthionine sulfoximine (BSO) [29]. Another group found that silencing either *ALOX15B or ALOXE3* prevented erastin-induced cell death, which supported the hypothesis that LOXs are required for ferroptosis initiated by class 1 inducers [12]. In addition, several LOX inhibitors were found to prevent erastin-induced ferroptosis in MEFs and pancreatic cancer cells [30,31]; however, as noted above, many LOX inhibitors also act as radical trapping antioxidants that suppress nonenzymatic propagation of lipid peroxidation. It has, however, been found that tumor cell ferroptosis is promoted by 15-lipoxygenase-catalyzed lipid peroxidation in cellular membranes [32].

The hypothesis that ALOX5 is involved in initiating ferroptosis was supported by the observation that deuteration at the 7 position of AA was protective against ferroptosis initiated by RSL3 [15]. Deuterium is heavier than hydrogen, and because of the primary deuterium isotope effect, removing a deuterium atom is significantly slower than removing a hydrogen atom to initiate peroxidation. As a consequence, PUFAs with deuterium at the bis-allylic carbons are unable to undergo peroxidation. Since the 7 position is the site of ALOX5 peroxidation, this result is consistent with a role for ALOX5. On the other hand, it is possible that the location of the 7 postion in the phospholipid membrane renders it more reactive to peroxidation overall so that substitutions at that position would have a larger effect on the rate of peroxidation. In summary, current data support a model in which both LOXs and nonenzymatic Fenton chemistry contribute to lipid peroxidation during ferroptosis.

## Where does lipid peroxidation take place?

**Fig 3:**
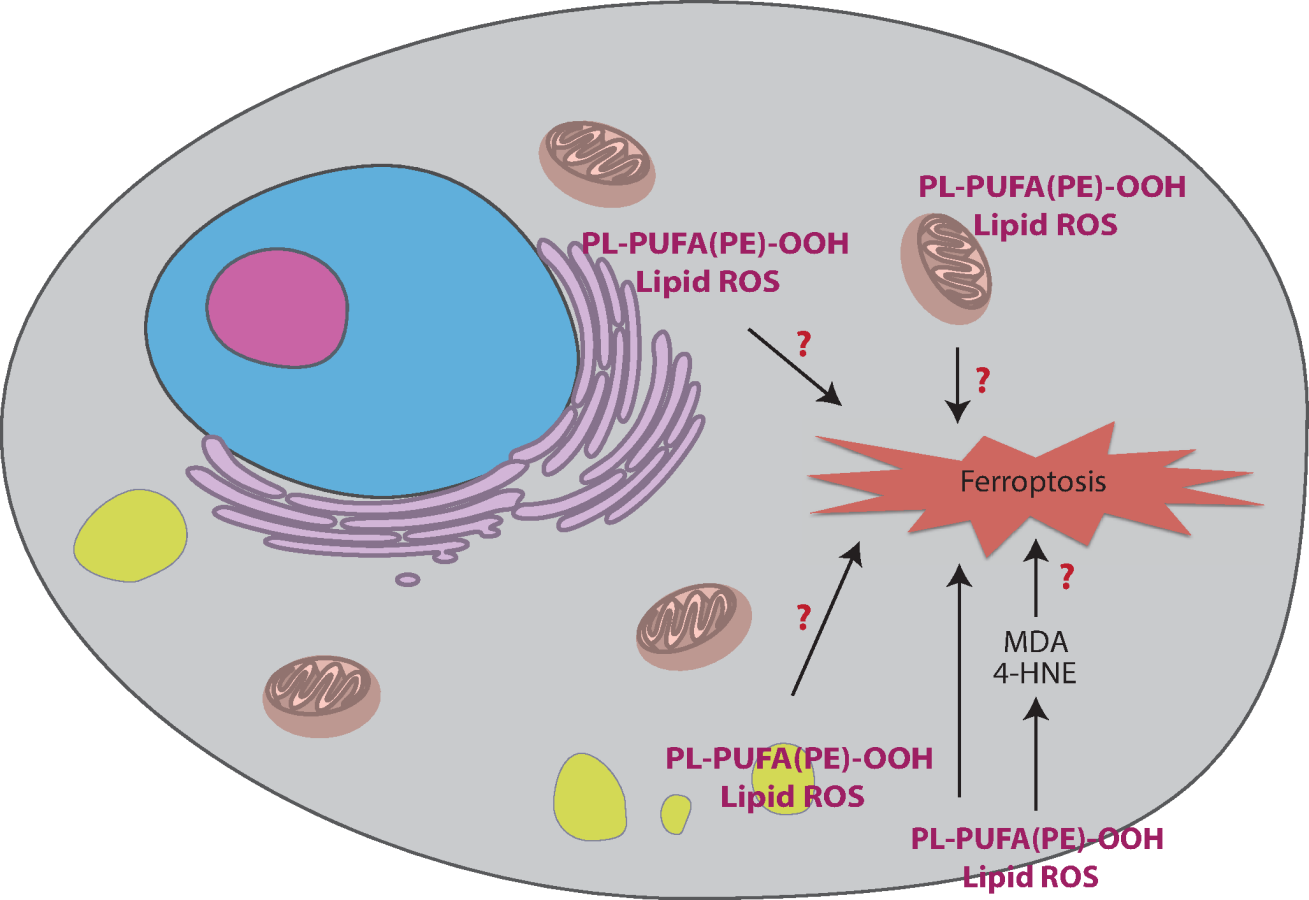
Subcellular model of the location of lipid peroxidation in ferroptosis. The red question marks represent the unsolved mysteries discussed in the article. 4-HNE, 4-hydroxynonenal; MDA, malondialdehyde; PL-PUFA(PE)-OOH, polyunsaturated-fatty-acid-containing-phospholipid hydroperoxides; ROS, reactive oxygen species.

Another important question is the location of lipid peroxidation during ferroptosis (Fig 3). A default assumption has been that lipid peroxidation occurs in the plasma membrane during ferroptosis because peroxidation of PUFA-PLs in the plasma membrane could compromise the integrity of cells. However, in addition to the plasma membrane, lipid peroxidation can also occur in other subcellular locations. Among these, mitochondria, ER, and lysosomes are three plausible candidates for the sites of lethal lipid peroxidation during ferroptosis.

Unlike apoptosis, ferroptosis in cancer cells doesn’t involve mitochondrial damage but is accompanied by alterations in mitochondrial morphology and intergrity [3]. Moreover, GPX4 has three isoforms located in the cytosol, nucleus, and mitochondria, and the nitroxide antioxidant XJB-5-131 targeted to mitochondria was discovered to be a more potent ferroptosis inhibitor than nontargeted nitroxides [33]. Thus, the hypothesis emerged that lipid peroxidation occurs in mitochondria during ferroptosis. However, direct evidence linking mitochondrial lipid peroxidation to ferroptotic cell death is still lacking. Recently, we generated mitochondria-deficient cells by introducing the E3 ligase Parkin and inducing mitochondria uncoupling with carbonyl cyanide m-chlorophenyl hydrazone (CCCP); this results in elimination of mitochondria through mitophagy [34]. We then treated the mitochondria-deficient cells with different ferroptosis inducers. The cells lacking mitochondria still underwent ferroptosis, indicating that the presence of mitochondria is not necessary for ferroptosis [35]. We also measured total ROS levels in mitochondria-deficient cells and in normal cells. As expected, we detected increased ROS in the presence of IKE, even in cells lacking mitochondria, albeit to a lesser extent compared to mitochondria-replete cells. These data suggest that lipid peroxidation does occur in mitochondria but is not required for ferroptosis.

The ER contains the largest pool of lipids in cells. Lipid peroxidation is thus likely to occur in the ER [36]. However, the relationship between ER lipid peroxidation and ferroptosis is still mysterious. ER stress and the unfolded protein response (UPR) might be related to ferroptosis. ER stress is caused by the accumulation of unfolded/misfolded proteins, which can be caused by a number a pathophysiological disturbances, including glucose deprivation, hypoxia, aberrant Ca^2+^ regulation, and viral infection. [37,38]. ER stress activates a complex signaling network—termed the UPR—to reduce ER stress and restore homeostasis [39].

Inhibition of system x_c_^−^ by erastin and the clinically approved anticancer drug sorafenib triggers some aspects of ER stress as well as ferroptosis, indicating a possible link between ER stress and ferroptosis [40]. One research group proposed that ROS are a signal generated by ER stress and UPR activation, consistent with their observation that antioxidant treatment reduces oxidative stress and UPR activation [41]. Another study showed that ER oxidoreduction-1 (ERO1) can trigger ROS generation in the ER by oxidizing protein disulfide isomerase (PDI) using a flavin adenine dinucleotide (FAD)-dependent reaction [42]. ER stress, ROS generation, and ferroptosis are possibly related, but the mechanisms driving lipid peroxidation in the ER and its relationship to ferroptosis are still unclear.

Lysosomes are another subcellular compartment hypothesized to be involved in lipid peroxidation during the course of ferroptosis, supported by the fact that ferrostatins accumulate in lysosomes using stimulated Raman scattering (SRS) microscopy [35]. It has been found that ROS are constitutively generated in lysosomes, using fluorescent ROS sensors [43]. Moreover, treatment with bafilomycin A1 (a lysosomal ATPase inhibitor), ammonium chloride (which neutralizes acidic organelles such as lysosomes), or PepA-Me (a lysosomal aspartyl protease inhibitor) suppressed ferroptosis induced by erastin and RSL3 and decreased lysosomal ROS and ferroptotic cell death–associated ROS. These studies suggest the possible involvement of lysosomal ROS in ferroptosis [43].

However, it was recently found that preventing accumulation of ferrostatins in lysosomes by reducing the extent of molecular trapping via the lysosomotropic effect improved the potency of ferroptosis inhibitors, which indicates that lysosomes are not critical for ferroptotic suppression [35]. The discovery of the iron-dependent lethal compound ironomycin that localizes to lysosomes [44] suggests that additional studies concerning the role of lysosomes in ferroptosis may be needed to evaluate the relationships between lysosomal lipid ROS and cell death.

## How does lipid peroxidation lead to ferroptotic cell death?

Another important open issue is what specifically during the course of lipid peroxidation leads to ferroptotic cell death. In other words, is the execution mechanism of ferroptosis through damage to specific membranes or through downstream reactive products? Membrane lipid peroxidation substantially alters the physical properties of lipid bilayers in terms of disrupted ion gradients, decreased membrane fluidity, slower lateral diffusion, and increased membrane permeability [45–48]. One hypothesis is that peroxidized phospholipids can reorient themselves and protrude into the aqueous phase, resulting in decreased membrane thickness and in macrophage recognition [47,49]. Recent molecular dynamics studies suggest that during ferroptosis, membrane thinning drives a vicious cycle of increasing access by oxidants, ultimately resulting in increasing membrane curvature and membrane damage through micelle formation [50].

Another hypothesis is that formation of protein-based pores is involved in ferroptosis, resulting in loss of ionic homeostasis [51]. Oxidized PUFA fragments have been hypothesized to destroy membranes and release toxic reactive fragments into cells, which might interfere with other cellular processes [52]. Incorporation of long PUFAs into membranes by ACSL4 sensitizes cells to ferroptosis [21]. Another group found that oxidation of ER-associated compartments occurs primarily on phosphatidylethanolamines (PEs) and suppression of acyls-arachidonyl and adrenoyl (AdA) esterification into PEs by inhibition of ACSL4 could prevent ferroptotic cell death [36].

Lipid peroxidation of PUFAs produces a wide variety of oxidation products. Lipid hydroperoxides (LOOHs) are the initial products of peroxidation. Secondary products are aldehydes, among which malondialdehyde (MDA) and 4-hydroxynonenal (4-HNE) are most abundant and exhaustively studied [19]. MDA is produced from decomposition of AAs and larger PUFAs via enzymatic and nonenzymatic pathways.

The enzymatic process has been extensively studied, whereas little is known about the nonenzymatic generation of MDA. MDA is the most mutagenic product of lipid peroxidation because of its capability to react with primary amines on proteins and DNA to form crosslinked adducts [53]. Moreover, excessive MDA generation within cells is associated with major human diseases such as Alzheimer disease, cancer, cardiovascular disease, diabetes, and Parkinson disease [54]. Decomposition of AAs and longer PUFAs generates 4-HNE [55]. This highly reactive product of lipid peroxidation contains 3 functional groups: (i) an electrophilic C = C double bond that is a Michael acceptor and that forms covalent adducts with nucleophilic amino acids, (ii) an aldehyde that can form Schiff base adducts with primary amines, and (iii) a hydroxyl group that can be oxidized to an electrophilic ketone [56].

The electrophile 4-HNE has been widely studied as a signaling molecule that stimulates the cell cycle and cell proliferation and as a cytotoxic molecule that inhibits gene expression and promotes the development of disease [19]. Moreover, it has been proposed that 4-HNE can induce cell death by modulating several transcription factors, such as Nrf2 and peroxisome proliferator-activated receptors (PPARs), as well as other signaling pathways, such as cell-cycle regulators and caspases [57]. It has also been found that whether cells undergo apoptosis or necrosis depends on the cellular concentration of 4-HNE [58]. Finally, selection of erastin-resistant clones of DU-145 cells revealed that resistant cells had dramatic upregulation of *AKR1C* genes, which are involved in detoxifying aldehydes such as 4-HNE [40][59]. A recent study profiled the protein carbonylation events induced during ferroptosis, presumably through such electrophilic products of lipid peroxidation [60]. Further studies need to be carried out to define the significance of 4-HNE during ferroptotic cell death.

It is worth noting that lipid peroxidation has also been associated in the literature with other modes of cell death, including apoptosis, in some cases. There are two key points to note about these prior studies. First, apoptosis was, for many years, considered synonomous with cell death, and many studies before the year 2000 simply termed cell death as apoptosis without any rigorous evaluation of the mode of cell death. Thus, these early studies that refer to apoptosis need to be reevaluated to examine the precise mode of cell death involved and whether indeed ferroptosis may have been induced. Second, in many cases, multiple cell death modes can be activated by pleiotropic triggers and stresses. Thus, the detection of markers of ferroptosis or apoptosis by themselves don’t illuminate the lethal death mechanism involved. In such cases, detection of lipid peroxidation during apoptosis may simply reflect a low-level stimulation of ferroptosis during apoptotic cell death. However, the possibility does remain that other models of cell death might be associated with nonlethal degrees of lipid peroxidation; such cases need to be carefully examined.

## Solving the mysteries

Some clues have emerged, pointing to possible solutions to these three mysteries. Regarding the mechanism of lipid peroxidation, as noted above, both Fenton chemistry and LOXs may contribute to ferroptosis. Iron metabolism and availability play key roles in both processes. It has been observed that decreased cellular iron levels suppress erastin lethality in *PHKG2*-silenced cells, indicating that phosphorylase b kinase γ 2 (PHKG2) regulates ferroptosis through regulation of labile iron [12]. Ferritinophagy, the autophagic degradation of ferritin, contributes to iron availability for Fenton chemistry and is involved in ferroptosis [61]. Additional links between iron metabolism and ferroptosis may help in clarifying the role of iron in ferroptosis. In addition, lipidomic studies may help to identify substrates of LOXs during ferroptotic cell death and to define which ferroptosis contexts primarily use LOXs versus Fenton chemistry.

Where lipid peroxidation occurs during ferroptosis is the second significant mystery. The plasma membrane, mitochondria, ER, and lysosomes are all candidates. As noted above, mitochondria-deficient cells revealed that mitochondria are not required for ferroptotic cell death. By reducing the extent of molecular trapping through the lysosomotropic effect, lysosomes were also shown to be unnecessary for erastin-induced and RSL3-induced cell death, as well as ferroptosis suppression by ferrostatins. Moreover, when SRS imaging was used to detect the localization of a diyne ferrostatin, no detectable signal was found in the plasma membrane. Therefore, we hypothesize that the ER might be a key subcellular location for ferroptosis, at least in the cancer cell lines studied in these experiments. Lipid peroxidation during ferroptosis may as well occur in multiple organelles. The vulnerability of each organelle to lipid peroxidation may be different because of differences in lipid compositions, iron storage, GSH level, LOX expression, and GPX4 localization. For example, mitochondria are rich in iron and GPX4. Depletion of mitochondria might change the cellular level of iron and GPX4 and regulate ferroptosis in other organelles. Defining the lipid composition of different organelles and how these lipids promote or suppress lipid peroxidation and ferroptosis may aid in solving this mystery.

Recently, a study reported that cyclic-di-adenosine monophosphate (c-di-AMP) in live gram-positive bacteria could interact with the innate sensor stimulator of interferon genes (STING) to mediate ER stress and induce mechanistic target of rapamycin (mTOR) inactivation, resulting in ER-phagy [62]. This study provides a strategy to induce ER-phagy in cells. To date, three proteins (family with sequence similarity 134 member B [FAM134B], SEC62, and reticulon-3 [RTN3]) have been identified as ER-phagy receptors in mammalian cells [63–65]; further elucidation of the components of this process may reveal links to ferroptosis. Since generation of mitochondria-deficient cells was possible using mitophagy, perhaps it will be possible to generate ER-deficient cells using ER-phagy. Moreover, as noted, ER stress, UPR, and subsequent ER-associated protein degradation (ERAD) are other key issues that may be linked to ferroptosis, and resolving their relationships may be informative. The ER membrane protein complex (EMC) might also function in both ERAD and ferroptotic cell death [66]. It is possible that ER stress is simply a consequence of GSH depletion during ferroptosis and does not contribute to the lethal mechanism, but more studies are needed to address this question.

How lipid peroxidation leads to ferroptosis is the third open question. Computational approaches such as molecular dynamics simulations may aid the study of membrane properties during ferroptosis. The secondary products of lipid peroxidation, such as MDA and 4-HNE, may also be good targets to examine for their roles during ferroptosis. Although these two aldehydes have been extensively studied, and excessive accumulation of 4-HNE has been shown to promote apoptosis and necrosis, their role in ferroptosis is unclear. By solving these mysteries of ferroptosis, we may discover new insights and therapeutic strategies for ferroptosis-related human diseases, such as numerous cancers and degenerative diseases.

## Abbreviations

2,2-BP: 2,2-bipyridyl
4-HNE: 4-hydroxynonenal
AA: arachidonic acid
ACSL4: acyl-CoA synthetase long chain family member 4
AdA: adrenoyl
ALOX: arachidonate lipoxygenase
BHT: butylated hydroxytoluene
BSO: buthionine sulfoximine
CCI_4_: carbon tetrachloride
CCCP: carbonyl cyanide m-chlorophenyl hydrazine
c-di-AMP: cyclic-di-adenosine monophosphate
CIL56: caspase-independent lethal 56
CoQ_10_: coenzyme Q_10_
COX: cyclooxygenase
CYP: cytochrome p450
DPI: diverse pharmacological inhibitor
D-PUFA: deuterated polyunsaturated fatty acid
EMC: endoplasmic reticulum membrane protein complex
ER: endoplasmic reticulum
ERAD: endoplasmic reticulum–associated protein degradation
ERO1: endoplasmic reticulum oxidoreduction-1
FAD: flavin adenine dinucleotide
FAM134B: family with sequence similarity 134 member B
Fer-1: ferrostatin-1
FIN56: ferroptosis inducer 56
FINO_2_: ferroptosis inducer endoperoxide
GPX4: glutathione peroxidase 4
GSH: glutathione
GSSG: glutathione disulfide
HMG-CoA: β-hydroxy β-methylglutaryl-CoA
HPETE: hydroperoxyeicosatetraenoic acid
IKE: imidazole ketone erastin
LOOH: lipid hydroperoxide
LOX: lipoxygenase
LPCAT3: lysophosphatidylcholine acyltransferase 3
MDA: malondialdehyde
MEF: mouse embryonic fibroblasts
ML162: Molecular Libraries 162
mTOR: mechanistic target of rapamycin
NCCD: Nomenclature Committee on Cell Death
PDI: protein disulfide isomerase
PE: piperazine erastin
PHKG2: phosphorylase b kinase γ 2
PPAR: peroxisome proliferator-activated receptor
PUFA: polyunsaturated fatty acid
PUFA-PL: polyunsaturated-fatty-acid-containing phospholipid
ROS: reactive oxygen species
RSL3: RAS-selective lethal 3
RTN3: reticulon-3
SQS: squalene synthase
STING: stimulator of interferon genes
UPR: unfolded protein response
